# From Employment Pressure to Entrepreneurial Motivation: An Empirical Analysis of College Students in 14 Universities in China

**DOI:** 10.3389/fpsyg.2022.924302

**Published:** 2022-07-12

**Authors:** Liping Yin

**Affiliations:** School of Cultural Industry and Tourism Management, Henan University, Kaifeng, China

**Keywords:** employment pressure, entrepreneurial values, entrepreneurial environment, necessity entrepreneurship, opportunity entrepreneurship, entrepreneurial motivation

## Abstract

Entrepreneurship is vital in solving the challenges involved in the employment of college students in China. The two viewpoints on the relationship between employment and entrepreneurship are the refugee effect and the entrepreneurial effect. However, the micro-mechanism of this association is yet to be comprehensively discussed. Based on the refugee effect and entrepreneurial effect, along with the entrepreneurial values as the mediating mechanism and the entrepreneurial environment as the moderating variable, this study establishes a theoretical model exploring the impact of employment pressure on necessity entrepreneurship and opportunity entrepreneurship of college students. Moreover, it selected 14 universities covering the eastern, central, and western regions of China. A total of 1,187 college students were surveyed anonymously using a standardized questionnaire. Then, SPSS 24.0 and Mplus 7.0 were employed to process and analyze the data, and the Structural Equation Modeling was established to test the hypothesis. The results demonstrated that employment pressure had a significant positive impact on college students’ necessity for entrepreneurship. Also, employment pressure indirectly affected necessity entrepreneurship and opportunity entrepreneurship through the mediating effect of entrepreneurial values. The entrepreneurial environment (a) positively moderated the relationship between employment pressure, necessity entrepreneurship, and opportunity entrepreneurship and (b) moderated the mediating effect of entrepreneurial values. That is, in a favorable entrepreneurial environment, entrepreneurial values have a stronger mediating effect. Starting from cognitive psychology, this study explored the micro-psychological mechanism of individual employment pressure that has influenced entrepreneurial motivation. It has enriched the existing literature on the entrepreneurship theory of college students, underlining that the hypothesis on refugee effect and entrepreneurial effect is also applicable at the micro-level. On top of that, it has provided a practical reference for the employment and entrepreneurship of current Chinese college students.

## Introduction

The two viewpoints on the relationship between employment and entrepreneurship are the refugee effect and the entrepreneurial effect. Amidst an economic depression, employment falls and workers find it difficult to secure a job that pays them a living wage. In this context, self-employment becomes relatively attractive and the number of people opting to start a business will increase. This phenomenon is known as the refugee effect (Aubry et al., 2015). Entrepreneurs with managerial or business acumen seize new opportunities to establish their businesses, enabling them to employ themselves and others, which is also known as the entrepreneurial effect (Emami Langroodi, 2021). According to the definition of Global Entrepreneurship Monitoring (GEM), necessity entrepreneurship is for people to make a living, while opportunity entrepreneurship involves entrepreneurial activities that develop and utilize discovered business opportunities or create them. Necessity entrepreneurship and opportunity entrepreneurship have a corresponding relationship with the refugee effect and entrepreneurial effect. As for the link between unemployment and entrepreneurship, the existing empirical research findings are inconsistent. Some researchers believe that a considerable number of unemployment-related problems promote entrepreneurial activities (Aubry et al., 2015; Li, 2018) but had no effect on the refugee effect (Apaydn, 2018). Kone et al. (2020) noted that if unemployment grows in neighboring regions, incentives for entering self-employment increase, implying the presence of the refugee effect. Since these studies are mostly based on macroeconomics, the heterogeneity of micro and individual factors is often ignored. Nevertheless, individual heterogeneity, such as educational level, plays a central role in explaining both the refugee effect and the entrepreneurial effect. Micro studies have also confirmed that the educational attainment of entrepreneurs has a significant impact on the growth rate of new enterprises (Backman et al., 2020).

**FIGURE 1:**
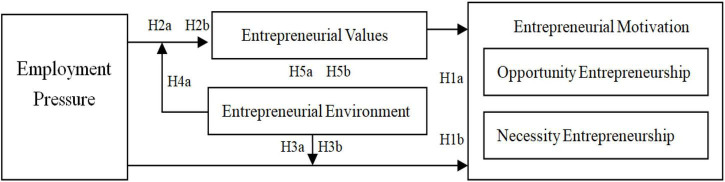
Research model.

Nowadays, the reality is that Chinese college students are facing severe employment problems. According to relevant data, there were 8.74 million college graduates in China in 2020, and the employment rate was 90%. In other words, nearly 900,000 college graduates did not find suitable jobs at that time. Of the 9.09 million college graduates in 2021, less than 90% found jobs. While the employment rate of college graduates continues to decrease, the proportion of those who choose to start their businesses is on the rise. In 2020, 820,000 college graduates started businesses, which was 11% higher than in 2019, and in 2021, 1.45 million college graduates founded their businesses, 16% higher than in 2020. Does this prove that the refugee effect and the entrepreneurial effect also apply to college students? What is the internal mechanism? Based on these questions, this study attempts to explore the relationship mechanism between employment and entrepreneurship from a micro perspective and establish a theoretical model of employment pressure influencing college students’ entrepreneurial motivation through entrepreneurial values (Figure 1). In this context, the research questions are as follows:

(1) Does the refugee effect or entrepreneurial effect exist among college students? In other words, does the current employment pressure, to some extent, enhance the necessity entrepreneurship or opportunity entrepreneurship of college students?
(2) If the above effects are true, what is the mediating mechanism of employment pressure influencing entrepreneurial motivation?
(3) Can an entrepreneurial environment promote the relationship between college students’ employment pressure and entrepreneurial motivation?

Overall, this research argues that an individual’s employment pressure initiates the process involved in building their entrepreneurial motivation. By integrating the perspectives of refugee effect and entrepreneurial effect, this study aims to examine the impacts of exogenous elements (e.g., COVID-19, entrepreneurial environment) and endogenous factors (e.g., employment pressure, entrepreneurial values) on college students from 14 Chinese universities. Additionally, it seeks to evaluate the micro psychological mechanism of the formation of individual entrepreneurial motivation, as well as verify the similarities and differences between the refugee effect and entrepreneurial effect among highly educated people. Finally, it discusses the role of the entrepreneurial environment in the formation of individual entrepreneurial motivation during a crisis.

## Literature Review and Hypotheses

### Employment Pressure and Entrepreneurial Motivation

Considering the impact of COVID-19, China’s economy has suffered a severe downturn, and the employment environment has become increasingly complex. The grave unemployment situation has led to greater employment pressure among today’s college students (Jin et al., 2020). According to the Panel Study of Chinese University Students (PSCUS), the COVID-19 pandemic has negatively impacted the employment of new graduates in several aspects, resulting in interrupted interviews, reduced employment rates, increased employment pressure, and pessimistic economic expectations for the future (Li, 2020). The economist Joseph Schumpeter argued that such crises were the “seedbeds of innovation and entrepreneurship.” In this case, the crisis could be perceived as a turning point for new perspectives and ideas, including new business opportunities (MacKenzie et al., 2020). According to the refugee effect, entrepreneurship increases during an economic depression while jobs are difficult to secure. Based on the entrepreneurial effect, labor costs fall when unemployment increases. Under the active promotion of the government’s entrepreneurship policy, entrepreneurs with business expertise will seize the opportunity and set up their enterprises accordingly. It can be seen that both the refugee effect and entrepreneurial effect consider that employment difficulties could promote entrepreneurial activities, and scholars have confirmed both hypotheses. For example, Thurik et al. (2008) studied the data on the unemployment rate and self-employment rate of 23 Organization for Economic Cooperation and Development (OECD) member countries from 1974 to 2002 and found that both the refugee effect and entrepreneurial effect played a role in OECD member countries, but the effect of the latter was far more significant than the former. Meanwhile, Apaydn (2018) examined the relationship between unemployment and entrepreneurship in Turkey. It turned out that when the rate of entrepreneurship rises, unemployment falls. This effect is largely driven by the entrepreneurial effect and not by the refugee effect. Vegetti and Adascalitei (2017) investigated the data of 25 EU member states from the individual and national levels and determined that high unemployment rates caused by an economic crisis have a refugee effect. However, it is mainly driven by workers who cannot find ideal jobs. Desai et al. (2021) compared the entrepreneurial tendencies of local residents and immigrants and found that immigrants are more willing to engage in self-employment because they receive less remuneration for the same type of work. In other words, economic factors resulted in their necessity for entrepreneurship. Fu (2010) noted the significant positive correlation between unemployment and entrepreneurial activities in China. That is, rising unemployment leads to an increase in entrepreneurial activities, which is predominantly self-employment. In other words, the refugee effect plays a significant role. To sum up, the problem of employment or unemployment can bring about the refugee effect and trigger the entrepreneurial effect.

In terms of entrepreneurship types, Murnieks et al. (2020) considered that the different pursuits of economic interests shaped the distinct forms of entrepreneurship. Some entrepreneurship is meant to meet one’s survival needs, such as street vendors. Another may be driven by various motives like economics or starting a second business. GEM believes that entrepreneurship consists of necessity entrepreneurship and opportunity entrepreneurship. The Ministry of Human Resources and Social Security (MOHRSS) of the People’s Republic of China investigated youth entrepreneurship activities in China. It found that, regarding entrepreneurial motivation, the Chinese youth are driven by either necessity or opportunity. Opportunity entrepreneurship refers to entrepreneurial activities carried out by entrepreneurs because they see potential business opportunities, while necessity entrepreneurship involves entrepreneurial activities executed by entrepreneurs to make a living (Fairlie and Fossen, 2017). In this way, the refugee effect is primarily driven by necessity entrepreneurship, while the entrepreneurial effect is mainly motivated by opportunity entrepreneurship. Several scholars have substantiated the relationship between employment and necessity entrepreneurship. For example, shortly after the COVID-19 outbreak, Li (2020) surveyed about 17,000 current students and 5,000 graduates from 19 institutions of higher learning in China. The study discovered that the proportion of college students who started a business after failing to find a satisfactory job 3 months after graduation was 3 percentage points higher than before the pandemic. It highlighted that, in the face of severe employment pressure, some of the graduates opted to start a business. Kuang et al. (2021) investigated more than 300 entrepreneurial cases in China and determined that although 62.3% of young people in poor areas had higher goals to pursue while running businesses, 36.8% were necessity entrepreneurs. They mainly started businesses to employ themselves and support their families. A meta-analysis on entrepreneurial motivation showed that material benefit is the main drive behind individual entrepreneurship. More specifically, the primary goals of entrepreneurs who just entered a new market are to generate income and make a living (Murnieks et al., 2020). Some scholars have also paid attention to the relationship between employment and opportunity entrepreneurship. For example, Jafari-Sadeghi et al. (2019) found that despite the economic recession caused by the COVID-19 pandemic and its evident impact on employment, it provided entrepreneurs with business opportunities. Even in developing countries, some entrepreneurs were eager to try. Xiong et al. (2021) surveyed 4,588 young entrepreneurs in China and highlighted that from the primary motivation of entrepreneurship, survival-based motivation accounted for 25.2%, the pursuit of wealth for 17.9%, and other motivations for 56.9%. Kariv et al. (2022) observed that entrepreneurs were flexible in responding to shocks during the pandemic, and those female entrepreneurs were motivated to start their businesses because of financial opportunities. Based on the above analysis, it can be inferred that it is more challenging for college students to secure their ideal jobs during a pandemic. Another portion of the college students in that study may weigh unsatisfactory jobs against potential business opportunities, resulting in opportunity entrepreneurship. Therefore, the following hypotheses are proposed:

**H1a:** There is a positive relationship between employment pressure and necessity entrepreneurship.

**H1b:** There is a positive relationship between employment pressure and opportunity entrepreneurship.

### The Mediating Effect of Entrepreneurial Values

Entrepreneurial values refer to an entrepreneur’s understanding of the importance of entrepreneurial goals, as well as the criteria necessary for evaluating and choosing relevant opportunities based on their needs (Sheng, 2020). Additionally, these values influence how people perceive entrepreneurial decisions and ultimately take action (Gorgievski et al., 2018). From the micro and individual levels, college students’ entrepreneurial values are relatively specific, reflected in the establishment of economic organizations, economic gains, and other aspects. As an explicit expression of entrepreneurial values, it is also a crucial force in promoting entrepreneurial behavior (Qiu, 2017). Entrepreneurial values are not innate and are developed gradually based on one’s specific conditions and foundations. Moreover, an individual’s social environment is fundamental to the formation of entrepreneurial values (Xu et al., 2020). These values reflect the social cognition that promotes the capacity of individuals to adapt to their environment and are a means of employment endowed with value (Hueso et al., 2020). Existing literature has covered the influence of a special environment on entrepreneurial values and found that upon encountering a disaster, entrepreneurs actively establish enterprises for boosting economic recovery and helping others overcome their economic difficulties (Dutta et al., 2021). For example, Gorgievski et al. (2018) determined that college students with entrepreneurial intentions would enhance their entrepreneurial values amidst an uncertain economic environment. To some extent, these studies confirm that in an economic depression and when employment is difficult, people’s recognition and pursuit of entrepreneurship are stimulated. For college students, due to the COVID-19 pandemic, coupled with the increasing number of graduates annually, their job search has become increasingly fierce and cutthroat, while the employment rate has continued to decline. Subsequently, it has become steadily difficult to find a satisfactory job. On top of that, it can be predicted that employment pressure is likely to make college students develop new cognition and views on the value and significance of entrepreneurship, that is, it may promote the formation and improvement of their entrepreneurial values.

As a type of professional value, entrepreneurial value is an individual’s judgment of the significance of entrepreneurship. It reflects their career attitude, motivation, and tendency, guiding and regulating individual entrepreneurial goals and behaviors (Liu et al., 2019). Numerous studies have established that entrepreneurial values largely determine the entrepreneurial motivation of individuals (Martínez-González et al., 2019) and continue to play a vital role in entrepreneurial activities (Hueso et al., 2020). In entrepreneurial activities, entrepreneurial values inspire individuals to take action and pursue their important and desired goals, stimulating the internal motivation of college students and then fueling their actual entrepreneurial behaviors (Xu et al., 2020). Fayolle et al. (2014) observed that when entrepreneurs have a strong need to realize their goals through entrepreneurship, they tend to have stronger entrepreneurial intention and motivation. For instance, according to Eyel et al. (2020), the entrepreneurial values of college students majoring in business management significantly impact entrepreneurial motivation. Santos et al. (2021) found that humane entrepreneurship can drive individual entrepreneurial motivation and has a greater effect on opportunistic entrepreneurial motivation. Furthermore, Xu et al. (2020) confirmed that entrepreneurial values have a significant mediating role between the entrepreneurial environment and the entrepreneurial intention of college students. Overall, the above analysis underscores that employment pressure promotes the formation of entrepreneurial values in universities to a certain extent, and correct entrepreneurial values significantly affect entrepreneurial motivation. Based on this, the following hypotheses are proposed:

**H2a:** Entrepreneurial values mediate the positive relationship between employment pressure and necessity entrepreneurship.

**H2b:** Entrepreneurial values mediate the positive relationship between employment pressure and opportunity entrepreneurship.

### The Moderating Effect of the Entrepreneurial Environment

The entrepreneurial environment accompanies the entire process of entrepreneurship. It is a general term for external factors that influence entrepreneurial activities, including direct and indirect environmental factors. The former comprises talent, capital, and site, while the latter encompasses policy measures, social culture, and social and economic conditions (Tang et al., 2011). The entrepreneurial environment used in this study mainly refers to the institutional environment, involving the formal system, such as the legal and tax systems and various regulatory measures, and the informal system, such as cultural values and social norms. Moreover, entrepreneurial activities depend on the interactions between the individuals and the environment (Lim et al., 2017). When the entrepreneurial environment is rich in both information and resources necessary for entrepreneurship, entrepreneurs will become more confident in successfully converting business opportunities into entrepreneurial actions and generating profits (Dutta et al., 2021). Fernández-Serrano et al. (2018) studied the relationship between national economic level, entrepreneurial culture, and entrepreneurial motivation. Their research underlined that, in developing countries experiencing economic recession, if the government can provide good entrepreneurial conditions, it can motivate the entrepreneurial spirit and ambitions of the entrepreneurs. Ibrahim and Masud (2016) pointed out that a relaxed entrepreneurial environment can increase the probability of opportunities being discovered, and the supportive attitude of the society toward entrepreneurship and entrepreneurial activities can enable entrepreneurs to convert the newly identified business opportunities into new entrepreneurial activities. As an illustration, Zhao and Wang (2021) ascertained that the three perspectives of entrepreneurial environment, policy environment, and market environment positively impact the entrepreneurial motivation of farmers. In this case, the policy environment has the greatest influence on the farmers’ opportunity entrepreneurship. Dong et al. (2012) believed that the rising unemployment rate and increasing employment pressure would reduce individual employment opportunities and wages. Under such circumstances, if the government adopts tax cuts or subsidies to stimulate self-employment, it will inevitably encourage more workers to choose self-employment. Meanwhile, college students lack entrepreneurial and work experience, and the external environment is more likely to affect their entrepreneurial motivation. When the external environment encourages and supports entrepreneurship, their entrepreneurial initiative and enthusiasm become easily mobilized. Therefore, the following hypotheses are proposed:

**H3a:** Entrepreneurial environment positively moderates the relationship between employment pressure and necessity entrepreneurship. Under a favorable entrepreneurial environment, the employment pressure will make college students more likely to start necessity entrepreneurship.

**H3b:** Entrepreneurial environment positively moderates the relationship between employment pressure and opportunity entrepreneurship. In a favorable entrepreneurial environment, the employment pressure will promote college students and make them more motivated for opportunity entrepreneurship.

Social cognition theory emphasizes that individuals acquire information from the external environment and construct self-cognition and behavior to keep it consistent with the external environment. As a special set of values, entrepreneurial values are personal attitudes and views toward entrepreneurship, including the personal understanding of entrepreneurial goals and judgment and choice concerning entrepreneurial behavior (Fayolle et al., 2014). College students have limited entrepreneurial and work experience and their entrepreneurial values are mainly influenced by the external environment, such as school, family, and society. In the context of entrepreneurship, an encouraging and supportive environment affects the attitudes and views of undergraduate students. Through qualitative research, Liu (2019) determined that the transformation of the entrepreneurial values of most college students occurred in the first 2 years after starting a business and was accompanied by landmark events. Additionally, the transformation may be influenced by two or more factors at the same time. Among them, entrepreneurial environment and entrepreneurial activities are the vital factors affecting the transformation of the entrepreneurial values of college students. For this group of people, a good entrepreneurial environment can positively shape their attitude and understanding of the value of entrepreneurial activities while promoting their positive entrepreneurial motivation (Ariyani, 2021). Therefore, this study speculates that, under the current severe employment pressure, college students have a lower probability of finding satisfactory jobs. Due to the influence of the positive entrepreneurial environment, these students are likely to re-examine the value and significance of entrepreneurship and form positive entrepreneurial values. Therefore, Hypothesis 4 is proposed as follows:

**H4:** Entrepreneurial environment positively moderates the relationship between employment pressure and entrepreneurial values. Under a favorable entrepreneurial environment, the employment pressure will promote the positive entrepreneurial values of college students.

Furthermore, the entrepreneurial environment moderates not only the relationship between employment pressure and entrepreneurial values but also the indirect effect of employment pressure on entrepreneurial motivation through entrepreneurial values. Based on this reasoning, we posit the following hypotheses:

**H5a:** Entrepreneurial environment moderates the mediating relationship between employment pressure and necessity entrepreneurship, such that entrepreneurial values have a stronger positive effect when the entrepreneurial environment is favorable.

**H5b:** Entrepreneurial environment moderates the mediating relationship between employment pressure and opportunity entrepreneurship, such that entrepreneurial values have a stronger positive effect when the entrepreneurial environment is favorable.

## Methodology

### Procedure

The data in this study were mainly collected using the questionnaire survey (platform is called “Wenjuanxing”), which was conducted from 1 to 18 March, 2022. This period was the “graduation season” for Chinese college students, and students were focused on finding employment. To make the sample more representative, the 14 universities selected covered the eastern, central, and western regions of China. To this end, the researcher first contacted the counselors, teachers, and student leaders in charge of student affairs of each university and explained the purpose and requirements of the survey. After agreeing to provide support, they explained the survey’s purpose and requirements to the respondents and sent the link of the questionnaire to the respondents who filled in the questionnaire through self-report and anonymously submitted it online upon completing it. For compensation, the respondents received a cash reward of 3 yuan. To minimize the deviation of the common method bias in data collection, we used the following methods: (1) All questionnaires should be anonymously completed and submitted online, thereby reducing the investigator’s concerns. (2) All scales in the questionnaire were adopted from mature scales developed by previous scholars, which were proven to have high reliability and validity. (3) The language of the questionnaire should be objective and neutral, and biased questions must be avoided. A pre-test was conducted to verify this.

### Sample

A total of 1,556 questionnaires were collected. With regards to screening the questionnaires, those with the same IP address, as well as with a shorter answer time than normal or incomplete answers or with similar options were considered invalid. After the stringent screening, 1,187 valid questionnaires were obtained, with an effective rate of 76.3%. Among the 1,187 respondents, 58.3% (*n* = 693) were females and 41.7% (*n* = 494) were males. Then, 48.7% were freshmen and sophomores, and 51.3% were juniors and seniors. Students majoring in management and economics accounted for 60.5%, while 39.5% majored in non-business administration. Among the respondents, 24% came from universities listed in Project 985, Project 211, and double first-class universities. More specifically, 31.4% were from regular undergraduate universities, and 44.6% were from vocational colleges. Among them, 23% came from urban areas and 77% from rural areas. On average, 26.7% of the respondents have family members with entrepreneurial experience. Overall, the sample distribution was suitable for the subsequent data analysis.

### Instrument

The questionnaire was written in Chinese. According to the standard “translation-back translation” procedure, these scales were translated from the original English version into Chinese, and then, translated back into English. After the constant revisions, 15 students were used as pretest subjects, and the Chinese version of the questionnaire was preliminarily tested to evaluate the scale’s reliability, validity, and usability. Based on the pretest results, the language used in the questionnaire was modified to make it more consistent with the interviewee’s perspective. All items were measured from 1 (very inconsistent) to 5 (very consistent).

The *Employment pressure* was subjected to the scale compiled by Chen and Jiang (2009), consisting of 6 items, including “Nowadays, there are so many graduates, and they face fierce competition for jobs,” and “The employment situation of college students is grim.”

The *Entrepreneurial values* were subjected to the scale compiled by Deng (2007), which comprised 6 items, including “Starting a business is a good way to solve the employment problem,” and “Starting a business is beneficial to both yourself and others.”

The *Entrepreneurial environment* was subjected to the scale assembled by Angulo-Guerrero et al. (2017), involving 5 items, such as “The government provides many preferential policies for entrepreneurship,” and “The examination and approval procedures for enterprise registration are simplified and convenient.”

As for entrepreneurial motivation, there are factors like necessity entrepreneurship and opportunity entrepreneurship. *Necessity entrepreneurship* was subjected to the scale compiled by Wang and Zhu (2010), containing 5 items, including “I will start a business because of poor job prospects,” and “I will start a business to improve my economic situation.” *Opportunity entrepreneurship* was subjected to the scale compiled by Chen and Ma (2020), consisting of 5 items, including “My biggest dream is to become an entrepreneur,” and “I can find good business opportunities and start-up projects.” By referring to the existing literature, gender, grade, major, school level, household registration, and a family’s entrepreneurial background were selected as control variables (Tang et al., 2018).

## Results

### Reliability and Validity Tests

Because the research conducted measurement at the same time point and adopted the method of student self-evaluation, it was necessary to further test for discrimination between these variables. This study adopted the Harman single factor test. According to the test results, the variance explanation percentage of the first common factor was 30.4%, which was lower than 40%, demonstrating the absence of any serious problem involving the common method deviation in this study. Then, Cronbach’s α coefficient was used to test the reliability of each variable. The findings demonstrated that the Cronbach’s α coefficients of the five variables, namely, employment pressure, entrepreneurial values, entrepreneurial environment, necessity entrepreneurship, and opportunity entrepreneurship were 0.88, 0.89, 0.84, 0.89, and 0.87, respectively, which were all greater than 0.7. This indicates that the internal variables are highly consistent and meet the measurement requirements. Then, the discriminant validity of each variable was tested. As shown in Table 1, compared with the other models, the five-factor model had the best fitting effect (χ^2^ = 734.01, *DF* = 179, *CFI* = 0.93, *TLI* = 0.92, *RMSEA* = 0.07, *SRMR* = 0.05), suggesting the high differential validity of each scale.

**TABLE 1:** Confirmatory factor analysis results of discriminant validity of variables.

Model	Chi-square	*df*	χ*^2^*/*df*	CFI	TLI	RMSEA	SRMR
One-factor (EP + EV + EE + OE + NE)	4241.16	189	22.442	0.502	0.453	0.19	0.161
Two-factor (EP + EE ‵ EV + OE + NE)	2829.63	188	15.051	0.671	0.642	0.151	0.113
Three-factor (EP ‵ EE ‵ EV + OE + NE)	1471.36	186	7.916	0.84	0.824	0.103	0.062
Four-factor (EP ‵ EV ‵ EE ‵ OE + NE)	1179.2	183	6.443	0.902	0.861	0.092	0.06
Five-factor (EP ‵ EV ‵ EE ‵ OE ‵ NE)	734.012	179	4.105	0.935	0.922	0.07	0.051

*EP, employment pressure; EV, entrepreneurial values; EE, entrepreneurial environment; OE, opportunity entrepreneurship; NE, necessity entrepreneurship.*

### Correlation Analysis

Subsequently, Pearson correlation analysis was employed to test the correlation among the variables. Table 2 details the mean, standard deviation, and correlation coefficient of each variable. Employment pressure was (a) positively correlated with entrepreneurial values (*r* = 0.182, *p* < 0.01) and (b) positively correlated with necessity entrepreneurship (*r* = 0.092, *p* < 0.01). Meanwhile, entrepreneurial values were (a) positively correlated with necessity entrepreneurship (*r* = 0.362, *p* < 0.01) and (b) positively correlated with opportunity entrepreneurship (*r* = 0.391, *p* < 0.01). Next, necessity entrepreneurship was positively correlated with opportunity entrepreneurship (*r* = 0.652, *p* < 0.01), indicating that subsequent hypothesis verification could be carried out.

**TABLE 2:** Mean, standard deviation (SD), and correlation coefficient of variables (*N* = 1187).

Variable	1	2	3	4	5	6	7	8	9	10	11
Gender											
Grade	0.06										
Major	0.047	–0.02									
University	0.034	−0.122**	0.033								
HR	–0.052	0.160**	–0.031	−0.204**							
FMEE	0.034	−0.100**	0.045	0.136**	−0.125**						
EP	0.062*	0.068*	–0.011	−0.071*	–0.01	–0.025					
EV	–0.03	−0.119**	–0.038	0.096**	−0.088**	0.004	0.182**				
EE	0.004	−0.202**	–0.003	0.131**	–0.056	0.061*	0.038	0.396**			
OE	−0.213**	−0.171**	–0.044	0.206**	–0.018	−0.67*	0.012	0.391**	0.252**		
NE	−0.171**	−0.154**	−0.065*	0.137**	–0.014	−0.121**	0.092**	0.362**	0.322**	0.652**	
Mean	1.351	2.013	1.91	2.141	1.313	1.692	3.961	3.467	3.487	2.271	2.778
SD	0.496	0.791	0.29	0.621	0.463	0.464	0.843	0.847	0.902	0.969	1.022

*** p < 0.001, ** p < 0.01, *P < 0.05.

*Male = 1, female = 2; Grades are divided into three categories, freshman and sophomore = 1, junior = 2, Senior year = 3; There are two types of majors: non-economics and management = 1, economics and management = 2; In terms of school category, universities listed in Project 985 and Project 211, and double first-class universities = 1, ordinary undergraduate university = 2, higher vocational college = 3; HR is household registration: rural = 1, urban = 2; FMEE is family members’ entrepreneurial experience, yes = 1, no = 2.*

*Source: Collated by author.*

### Hypothesis Testing

#### H1a and H1b Tests

Mplus 7.0 was utilized for data analysis in this study. Table 3 itemizes the test results. After controlling specific variables, namely, gender, grade, major, school level, household registration, and whether family members have entrepreneurial experience, employment pressure was found to significantly positively affect necessity entrepreneurship (β = 0.108, *p* < 0.001). This validated H1a. Additionally, employment pressure had a positive effect on opportunistic entrepreneurial motivation, but it was not significant (β = 0.037, *p* > 0.05). In this case, H1b was not verified. Then, employment pressure significantly positively impacted entrepreneurial values (β = 0.183, *p* < 0.001). Among the control variables, gender significantly negatively affected necessity and opportunity entrepreneurship, respectively (β = −0.177, *p* < 0.001) and (β = −0.211, *p* < 0.01), indicating that male students exhibited stronger entrepreneurial motivation than female students. On the effect of school type on necessity and opportunity entrepreneurship had the values (β = 0.090, *p* < 0.01) and (β = 0.172, *p* < 0.001), suggesting that students from higher vocational colleges had stronger entrepreneurial motivation than those from Projects 985 and 211 and double first-class universities. Family members’ entrepreneurial experience significantly negatively influenced necessity and opportunity entrepreneurship. The values (β = −0.130, *p* < 0.01) and (β = −0.077, *p* < 0.01) highlight that family members’ entrepreneurial experience had a positive impact on the two types of entrepreneurial motivation of college students. Finally, the other variables had no significant influence on the two types of entrepreneurial motivation.

**TABLE 3:** Direct effect result.

Variable	Estimate	S.E.	Est./S.E.	*P*-value
Gender	−0.177***	0.026	–6.773	0
Grade	–0.077	0.034	–2.279	0.052
Major	–0.042	0.026	–1.577	0.115
University	0.09	0.034	2.632	0.008
HR	0.022	0.027	0.814	0.416
FMEE	–0.13	0.026	–4.902	0
H1a	0.108***	0.028	3.873	0
H1b	0.037	0.028	1.31	0.19

***p < 0.001.

#### H2a and H2b Tests

Latent variable modeling was used to estimate the mediating effect of employment pressure on entrepreneurial motivation with Mplus 7.0. Setting bootstrap resampling to 5,000 times to test H2a. The test results are shown in Table 4. After controlling for gender, grade, major, school type, household registration type, and the entrepreneurial experience of family members, the coefficient for the indirect impact of employment pressure on necessity entrepreneurship through entrepreneurial values was 0.064 (*p* < 0.001). Also, the 95% bias-corrected confidence interval ranged from LLCI = 0.031 to ULCI = 0.101, excluding 0, proving that the mediating effect of entrepreneurial values between employment pressure and necessity entrepreneurship was established, thereby confirming H2a. Then, the same method was employed to examine the mediating effect of H2b entrepreneurial values on employment pressure and opportunity entrepreneurship. According to the findings, the coefficient for the indirect impact of employment pressure on opportunity entrepreneurship through entrepreneurial values was 0.091 (*p* < 0.01), and the 95% bias-corrected confidence interval ranged from LLCI = 0.044 to ULCI = 0.142, excluding 0. This verified that the mediating effect of entrepreneurial values between employment pressure and opportunity entrepreneurship was established, confirming H2b.

**TABLE 4:** The mediating role and moderating role.

Hypotheses	Estimate	S.E.	Est./S.E.	*P*-value	95% CI	
					Lower	Upper
H2a	0.064	0.018	3.54	0	0.031	0.101
H2b	0.091	0.025	3.639	0	0.044	0.142
H3a	0.111	0.038	2.9	0.004	−	−
H3b	0.178	0.036	4.904	0	−	−
H4	0.299	0.035	8.655	0	−	−

#### H3a\H3b and H4 Tests

The next step tested the moderating effect of the entrepreneurial environment. The test results are shown in Table 4. The entrepreneurial environment positively moderated the relationship (a) between employment pressure and necessity entrepreneurship with a moderating effect of 0.178 (*p* < 0.001), and (b) between employment pressure and opportunity entrepreneurship with a moderating effect of 0.111 (*p* < 0.01). These findings verified both H3a and H3b, showing that a favorable entrepreneurial environment can promote a positive relationship between employment pressure and entrepreneurial motivation. However, the entrepreneurial environment had a greater moderating effect on the relationship between the employment pressure and the necessity entrepreneurship. Then, the same method was adopted to test the moderating effect of the entrepreneurial environment on the relationship between employment pressure and entrepreneurial values. The test results showed that the moderating effect of the entrepreneurial environment on the relationship between employment pressure and entrepreneurial values was 0.299 (*p* < 0.001), verifying H4. This implies that although the employment pressure increases in a good entrepreneurial environment, it can lead college students to form stronger entrepreneurial values. Conversely, it reduces their entrepreneurial values.

#### H5a and H5b Tests

This study calculated the indirect effect of employment pressure on necessity and opportunity entrepreneurship through entrepreneurial values under different levels of entrepreneurial environment. The high and low entrepreneurial environment groups were divided by means of mean plus or minus one standard deviation and the 95% bias-corrected confidence interval. Table 5 presents the test results. (1)When the entrepreneurial environment was favorable, the mediating effect of entrepreneurial values on the relationship between employment pressure and necessity entrepreneurship was 0.109 (*p* < 0.05). Also, the 95% bias-corrected confidence interval ranged from LLCI = 0.018 to ULCI = 0.187 (5,000 bootstrap resamples), which did not contain 0. When the entrepreneurial environment was unfavorable, the mediating effect of entrepreneurial values was 0.095 (*p* > 0.05). Therefore, the moderated mediation effect of entrepreneurial values is established, confirming H5a. (2) When the entrepreneurial environment was favorable, the mediating effect of entrepreneurial values on the relationship between employment pressure and opportunity entrepreneurship was 0.115 (*p* < 0.05), and the 95% bias-corrected confidence interval ranged from LLCI = 0.018 to ULCI = 0.193 (5,000 bootstrap resamples), which did not contain 0. When the entrepreneurial environment was not favorable, the mediating effect of entrepreneurial values was 0.101 (*p* > 0.05). Therefore, the moderated mediation effect of entrepreneurial values was established, confirming H5b.

**TABLE 5:** Results of moderated mediation analysis for EE.

Model		Estimate	S.E.	Est./S.E.	*P*-value	95% CI	
						Lower	Upper
H5a	High	0.109	0.041	2.294	0.022	0.018	0.187
	Medium	0.102	0.054	1.877	0.06	0	0.211
	Low	0.095	0.068	1.615	0.106	−0.019	0.254
H5b	High	0.115	0.044	2.23	0.021	0.018	0.193
	Medium	0.108	0.057	1.885	0.059	0	0.227
	Low	0.101	0.071	1.621	0.105	−0.021	0.261

**FIGURE 2:**
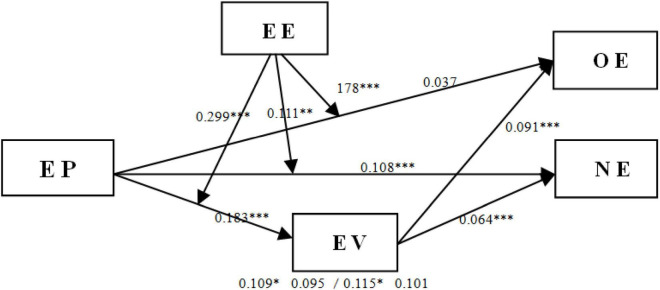
Findings for the structural equation model. Abbreviations: EP, employment pressure; EV, entrepreneurial values; EE, entrepreneurial environment; OE, opportunity entrepreneurship; NE, necessity entrepreneurship. ***p < 0.001, **p < 0.01, *p < 0.05.

Figure 2 illustrates the results of all hypothesis tests.

## Discussion

A total of 9 hypotheses were proposed in this study, but H1b was rejected, and the other 8 hypotheses were confirmed. H1b assumes that there is a positive relationship between employment pressure and opportunity entrepreneurship. The findings demonstrated that employment pressure had a positive effect on opportunity entrepreneurship (β = 0.037, *p* > 0.05), but it was not significant. This attests that opportunity entrepreneurship directly generated by college students due to employment pressure was minimal, which may be consistent with their individual characteristics, particularly most of them not having entrepreneurial experience and lacking the ability to grasp business opportunities. Simultaneously, due to the current economic recession in China, some companies have been in a dire situation, which may, to some extent, dampen the confidence of entrepreneurs (Giotopoulos et al., 2017). However, H1a (There is a positive relationship between employment pressure and necessity entrepreneurship) was confirmed. That is to say, when college students are faced with employment pressure, they become motivated to start a business to support their families or improve their lives. In China, college students are the hope of their families who often place great expectations on them, believing they will have a bright future after graduation. However, the COVID-19 pandemic has severely affected the employment prospects of undergraduate students. Under such circumstances, some may choose to start a business rather than settle for an unsatisfactory job. This study is also consistent with Li (2020) investigation of the entrepreneurship of Chinese college students, specifically that they are forced to start their own businesses.

Second, this study validated the mediating role of entrepreneurial values between employment pressure, necessity entrepreneurship, and opportunity entrepreneurship. However, entrepreneurial values had a greater mediating effect on employment pressure and opportunity entrepreneurship. Although the direct impact of employment pressure on opportunity entrepreneurship was not significant, it could affect opportunity entrepreneurship through the indirect role of entrepreneurial values. This assumed that when college students encounter various types of employment pressures, they will shift their attention from finding a job to starting a business. Moreover, they will augment their entrepreneurial values through their awareness and understanding of entrepreneurial activities. Once their positive entrepreneurial values are formed, these values will subtly influence their entrepreneurial motivation and establish a foundation for them to execute specific entrepreneurial activities in the future. In the existing empirical studies on the relationship between entrepreneurship and employment, most started from economic theories and macro data, which aimed to test the driving effect of entrepreneurship on employment. In addition, both the refugee effect and entrepreneurial effect have been indeed theoretical hypotheses proposed by scholars after the appearance of the phenomena. Hence, they do not reveal the micro mechanism of the relationship between unemployment and entrepreneurship. Under the impact of the COVID-19 pandemic, China’s unemployment rate has continued to rise, and the employment rate has generally declined. Consequently, increased employment pressure takes place among undergraduate students. In view of this specific situation, this study explored the micro mechanism of the refugee effect and entrepreneurial effect from the perspective of the changes in cognition, psychology, and motivation of college students. It also investigated the psychological changes of how college student entrepreneurs convert employment pressure into entrepreneurial motivation, so as to generate new evidence and ideas for the discussion of the relationship between employment and entrepreneurship.

Third, this study verified that the entrepreneurial environment (a) positively moderated the relationship between employment pressure and both opportunity entrepreneurship and necessity entrepreneurship, and (b) had a greater moderate effect on the relationship between employment pressure and opportunity entrepreneurship. In other words, when college students are in a positive entrepreneurial environment, they can convert the employment pressure into their motivation for entrepreneurship, forming positive entrepreneurial values and generating a strong motivation for opportunity and necessity entrepreneurship. Since business opportunities are closely related to the entrepreneurial environment, when the government implements more policies and measures to promote entrepreneurship, entrepreneurs can often identify business opportunities and attain entrepreneurial motivation (Fernández-Serrano et al., 2018). It is worth noting that the entrepreneurial environment can also ultimately affect both necessity entrepreneurship and opportunity entrepreneurship by moderating the mediating effect of entrepreneurial values. This substantiates that an unfavorable environment often becomes the soil for entrepreneurship and innovation to grow (MacKenzie et al., 2020). Equally important, unfavorable environments often inspire some people with an entrepreneurial spirit, making them more certain of the significance of entrepreneurship to social and economic development and the improvement of national life (Gorgievski et al., 2018; Dutta et al., 2021), resulting in a strong entrepreneurial motivation. Aubry et al. (2015) proposed to study the relationship between unemployment and entrepreneurship from the perspectives of the entrepreneurship system and environment, which is consistent with Xu et al.’s (2020) study that found entrepreneurial environment significantly promotes entrepreneurial motivation. This is also aligned with Ren et al.’s (2022) research on the current entrepreneurial state of Chinese college students. The study revealed that under the promotion of government policies like “entrepreneurship-driven employment” and “mass entrepreneurship and innovation,” college students have become the most active group in entrepreneurial activities. Among the Chinese entrepreneurs, the population aged 18–34 accounted for 44.39%.

### Theoretical Implications

First, this study discussed the mechanism of refugee effect and entrepreneurial effect from the micro and individual level and proved that the current entrepreneurial activities of Chinese college students presented the refugee effect, but the entrepreneurial effect was not significant. The two viewpoints about the relationship between employment and entrepreneurship are the refugee effect and the entrepreneurial effect. Scholars have distinct opinions about this. For example, Sheng (2017) analyzed the provincial panel data from 2000 to 2014 in China and found a weak entrepreneurial effect, but no refugee effect. Li (2018) investigated the four waves of entrepreneurship in China and believed that the refugee effect played a leading role in the first three, while the entrepreneurial effect was evident in the fourth. The reasons for the above contrast could be the differences in data selection, index acquisition, research objects, and surveyed regions. Therefore, the above findings did not fully reflect the real situation. An analysis based on the micro perspective of an individual can avoid these problems. Hence, through the data analysis at the individual and micro-level, this study confirms the different effects of the refugee effect and entrepreneurial effect among today’s Chinese college students, while expanding the research on refugee effect and entrepreneurial effect at the micro-level.

Second, this study opened the black box between employment pressure and entrepreneurial motivation and confirmed the important role of entrepreneurial values. In other words, the necessity entrepreneurship and opportunity entrepreneurship associated with the employment problem of college students differs from the refugee effect and entrepreneurial effect driven by economic interests. Moreover, college students are motivated to start a business primarily because they recognize the value of entrepreneurship. The discussion on the micro-mechanism between employment pressure and entrepreneurial motivation is valuable in the comprehensive understanding of the formation mechanism of the entrepreneurial motivation of college students, as well as improving the formation mechanism of refugee effect and entrepreneurial effect.

Third, this study combined exogenous elements (entrepreneurial environment) and endogenous factors (employment pressure, entrepreneurial values) that affect individual entrepreneurial motivation. It also discussed the influence of their interaction on individual entrepreneurial motivation. Then, it systematically considered the formation mechanism of college students’ entrepreneurial motivation and expanded the related research on entrepreneurial motivation. It also responded to Aubry et al.’s (2015) research that proposed to study the relationship between unemployment and entrepreneurship based on the entrepreneurial system and environment.

Fourth, this study examined the difference between necessity entrepreneurship and opportunity entrepreneurship by college students under special circumstances, further clarifying the different performances of refugee effect and entrepreneurial effect among the highly educated groups. In the existing literature, the research objects of refugee effect and entrepreneurial effect are mainly the unemployed, laid-off workers, and immigrants. Therefore, some scholars proposed that the level of human capital is a central factor influencing whether entrepreneurial activities are caused by refugee effect or entrepreneurial effect. It is also a fundamental variable that determines whether entrepreneurs option for necessity or opportunity entrepreneurship (Guo et al., 2021). This study responded to this point of view and unveiled that necessity entrepreneurship is larger than opportunity entrepreneurship among college students. However, in a favorable entrepreneurial environment, opportunity entrepreneurship is stronger than necessity entrepreneurship. In this context, this study complemented the role of one’s educational attainment and external environment in the refugee effect and entrepreneurial effect.

### Managerial Implications

From a practical point of view, the findings of this study will first help governments formulate targeted employment and entrepreneurship policies for college students and optimize the current entrepreneurial environment. Additionally, the entrepreneurial behavior of these students is either necessity- or opportunity-driven, and there are different public policy suggestions on necessity entrepreneurship and opportunity entrepreneurship for college student entrepreneurs. The policy for necessity entrepreneurship should stress the active support for entrepreneurs, where appropriate measures like start-up loans, tax exemptions, simplified applications, and review procedures should be adopted. Meanwhile, policies for opportunity entrepreneurship must encourage and support innovative and even risky entrepreneurial projects. The focus should be on supporting entrepreneurs in terms of the relevant information, technology, and platform, among several others. This could effectively solve the practical problems of college students, with distinct entrepreneurial motives, in their business activities or ventures. Equally essential, colleges and universities should strengthen the management of college students’ employment pressure, cultivate their entrepreneurial values, and guide them to regard entrepreneurship as a way of employment. As a result, they can objectively and comprehensively view and evaluate entrepreneurship. Simultaneously, institutes of higher education should cultivate the entrepreneurial skills and management expertise of undergraduate students (Lv et al., 2021), while encouraging more of them to partake in the entrepreneurship practice.

### Limitations and Future Research

There are several limitations to this study. First, the research data were mainly obtained from the self-reports of college students, which are susceptible to self-presentation biases, therefore, limiting their validity. In the future, the comprehensive use of macro data and micro and individual investigation can be considered, or the combination of quantitative and qualitative research can be adopted to improve the accuracy and external validity of the conclusion. Second, the examined sample was limited. As of September 2021, there were more than 2,700 ordinary colleges and universities in mainland China, but this study only covered 14. The insufficient survey sample size may lead to the limited applicability of these conclusion. Third, this study found that the refugee effect and entrepreneurial effect were more significant in higher vocational college students than in universities of Projects 985 and 211, as well as double first-class university students. The possible reason is that the graduates of top universities crowd out the employment opportunities of those from ordinary universities and higher vocational colleges. Consequently, the latter find it difficult to secure satisfactory jobs and are pushed to start their own businesses. It could also be that students from higher vocational schools have certain professional skills and advantages when it comes to starting a business. With the increasing employment pressure, they have become more willing to establish their own firms. Due to the limited number of universities surveyed, further verification and analysis are impossible at this point. In the future, the entrepreneurial motivation of college students from different types of universities can be compared to explore the differences in their entrepreneurial activities.

## Conclusion

This study mainly discussed the internal mechanism of the relationship between employment pressure and entrepreneurial motivation of college students in China. The investigation and analysis of 1,187 college students from 14 colleges and universities nationwide answered the three questions raised in the introduction. First, the refugee effect and entrepreneurial effect apply to current Chinese college students. When college students feel excessive employment pressure, they will have survival entrepreneurial motivation. Second, this study confirmed the intermediary role of entrepreneurial values in the relationship between employment pressure and entrepreneurial motivation. This illustrates that the phenomenon of college students’ entrepreneurial motivation caused by their employment problems differs from the refugee effect and entrepreneurial effect driven by unemployment. As highly educated workers, college students are not refugees who have to start a business due to unemployment. Instead, between settling for unsatisfying jobs and starting a business, they prefer the latter. Their entrepreneurial motivation is not only driven by material benefits but also includes gaining recognition and pursuing the value of entrepreneurship. Third, this study verified that the entrepreneurial environment played a positive role in the relationship between employment pressure and entrepreneurial motivation. When the entrepreneurial environment is encouraging, entrepreneurial pressure can significantly improve the necessity entrepreneurship and opportunity entrepreneurship of college students and can improve the latter to a greater extent. Meanwhile, the entrepreneurial environment can also promote the relationship between employment pressure and entrepreneurial values. Although college students face great employment pressure, a favorable entrepreneurial environment can promote the formation and improvement of their entrepreneurial values and ultimately form their entrepreneurial motivation.

## Data Availability Statement

The datasets presented in this study can be found in online repositories. The names of the repository/repositories and accession number(s) can be found in the article/supplementary material.

## Ethics Statement

The studies involving human participants were reviewed and approved by Ethics Committee of the Henan University. Written informed consent to participate in this study was provided by the participants.

## Author Contributions

LY designed the theoretical model and wrote the original manuscript. LY analyzed the data and improved the manuscript. LY contributed to writing review and editing. LY revised and supervised the entire work. LY contributed to the article and approved the submitted version.

## Conflict of Interest

The author declares that the research was conducted in the absence of any commercial or financial relationships that could be construed as a potential conflict of interest.

## Publisher’s Note

All claims expressed in this article are solely those of the authors and do not necessarily represent those of their affiliated organizations, or those of the publisher, the editors and the reviewers. Any product that may be evaluated in this article, or claim that may be made by its manufacturer, is not guaranteed or endorsed by the publisher.
